# Fixation of an esophageal stent using a novel re‐openable endoclip for a tracheoesophageal fistula

**DOI:** 10.1002/deo2.342

**Published:** 2024-03-02

**Authors:** Yo Kubota, Ryu Nishiyama, Masaya Sasaki, Yuta Sakabe, Kusutaro Doi, Hiroyuki Kitagawa, Hidehiko Kikuchi, Chika Kusano

**Affiliations:** ^1^ Department of Gastroenterology Hiratsuka Kyosai Hospital Federation of National Public Service Personnel Mutual Aid Associations Kanagawa Japan; ^2^ Department of Gastroenterology Kitasato University School of Medicine Kanagawa Japan

**Keywords:** endoclip, esophageal squamous cell carcinoma, esophageal stent, fixation, tracheoesophageal fistula

## Abstract

Although esophageal stenting is one treatment option as a palliative treatment for tracheoesophageal fistulas, serious complications are associated with stent migration. Some reports have described stent fixation using various devices to prevent stent migration. However, these have yet to be sufficiently examined. We performed esophageal stent fixation using the MANTIS Clip (Boston Scientific), a novel re‐openable endoclip. An 89‐year‐old man developed a tracheoesophageal fistula after radiotherapy for esophageal squamous cell carcinoma. Esophageal stenting was considered because the patient had difficulty with oral intake. However, the patient had a mild stenosis, which suggested stent migration. Therefore, we performed esophageal stent fixation by grasping the mouth side of the stent and the normal mucosa of the esophagus with the MANTIS Clip after placement of the stent. The esophageal stent closed the fistula, and the patient was able to take food orally. Upper gastrointestinal endoscopy performed 3 weeks after stenting showed residual MANTIS Clip and no evidence of stent migration. Esophageal stent fixation with MANTIS clips for tracheoesophageal fistulas may be an option to prevent stent migration.

## INTRODUCTION

Tracheoesophageal fistula is a complication of esophageal cancer treatment that significantly reduces the patient's quality of life owing to nutritional disturbance and cough. Tracheoesophageal fistula associated with esophageal cancer is often difficult to treat with surgery, and palliative care is often provided. Esophageal stenting is considered a treatment option for tracheoesophageal fistulas because it is a relatively simple and minimally invasive procedure.^1^ However, stent migration is a serious complication of esophageal stenting.^1^ A previous study has reported a stent migration rate of 20% in esophageal stenting for tracheoesophageal fistulas.^2^ The prevention of stent migration is important, as migration can lead to severe complications.^1^ There have been reports of stent fixation using various devices to prevent stent migration^3^, ^4^, ^5^; however, the evidence is insufficient. The MANTIS clip (Boston Scientific) is a novel endoclip with strong tissue grasping power due to the presence of anchor prongs. Moreover, the re‐openable clip allows for precise tissue regrasping. Here, we report a case of esophageal stent fixation using the MANTIS clip in a tracheoesophageal fistula.

## CASE REPORT

**FIGURE 1:**
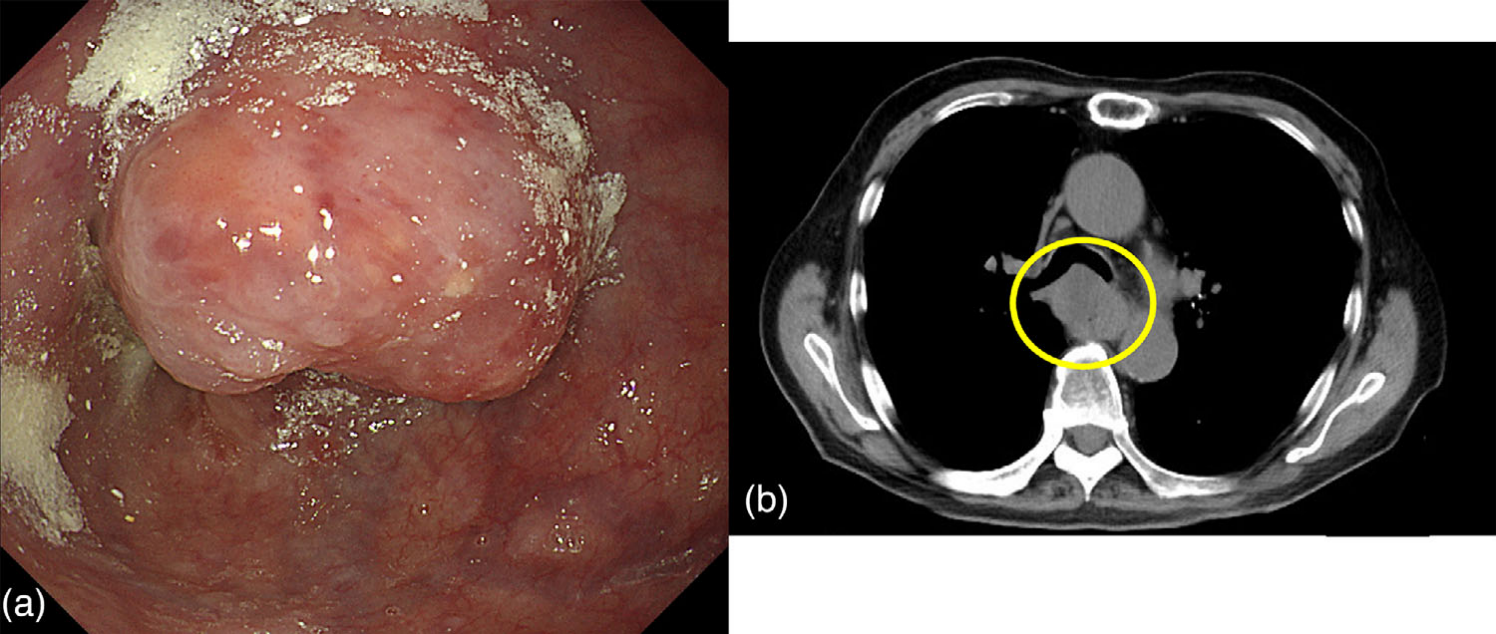
**Diagnosis of esophageal squamous cell carcinoma**. (a) Upper gastrointestinal endoscopy shows an elevated lesion with the upper wall of the mid‐thoracic esophagus as its main locus. (b) Computed tomography shows the tumor in contact with the bronchus (yellow circle).

**FIGURE 2:**
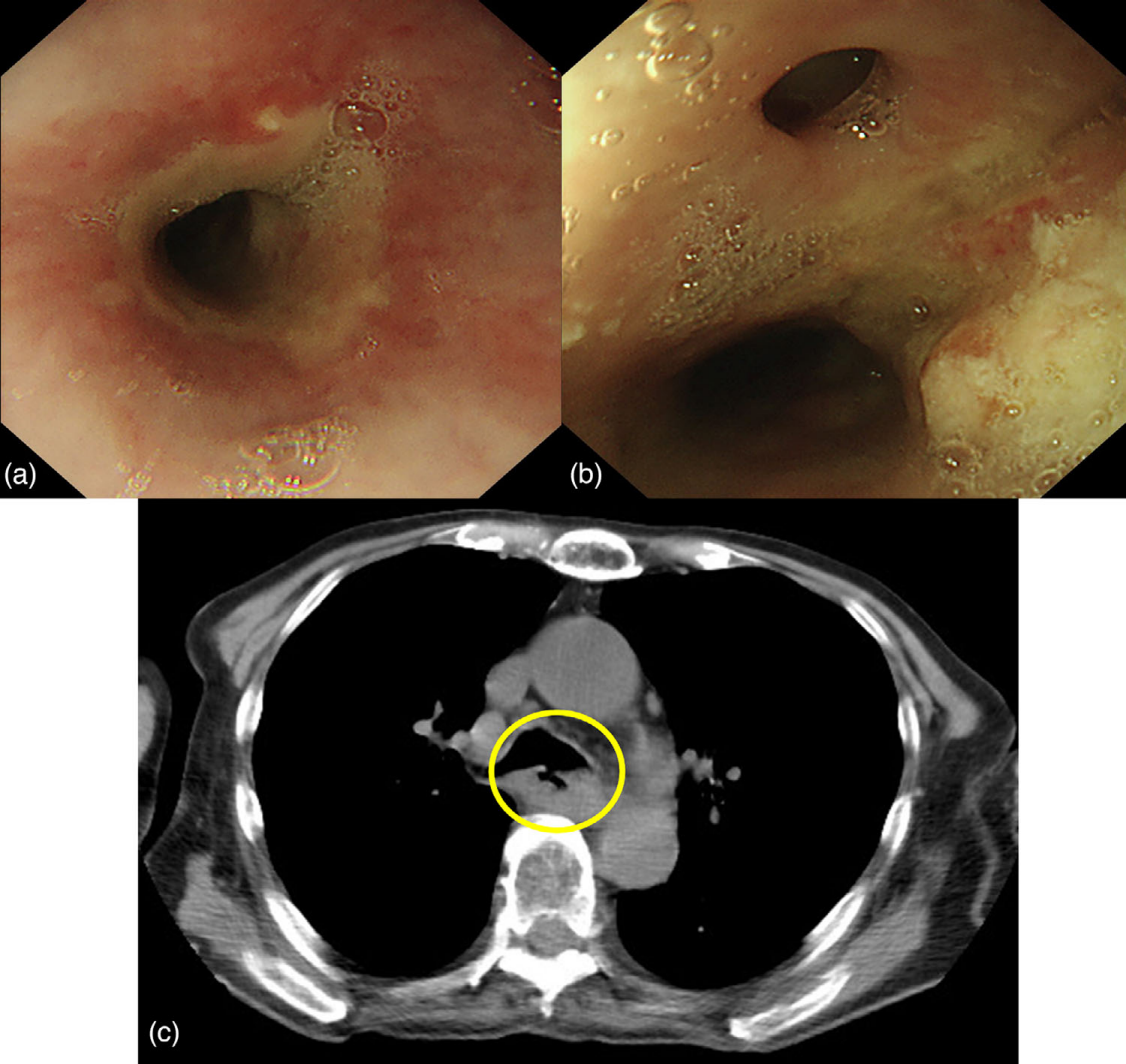
**After radiotherapy for esophageal squamous cell carcinoma**. (a) Upper gastrointestinal endoscopy shows a complete response to treatment for esophageal squamous cell carcinoma. (b) The fistula is confirmed in the middle esophagus. (c) Computed tomography shows esophageal and bronchial traffic (yellow circle).

**FIGURE 3:**
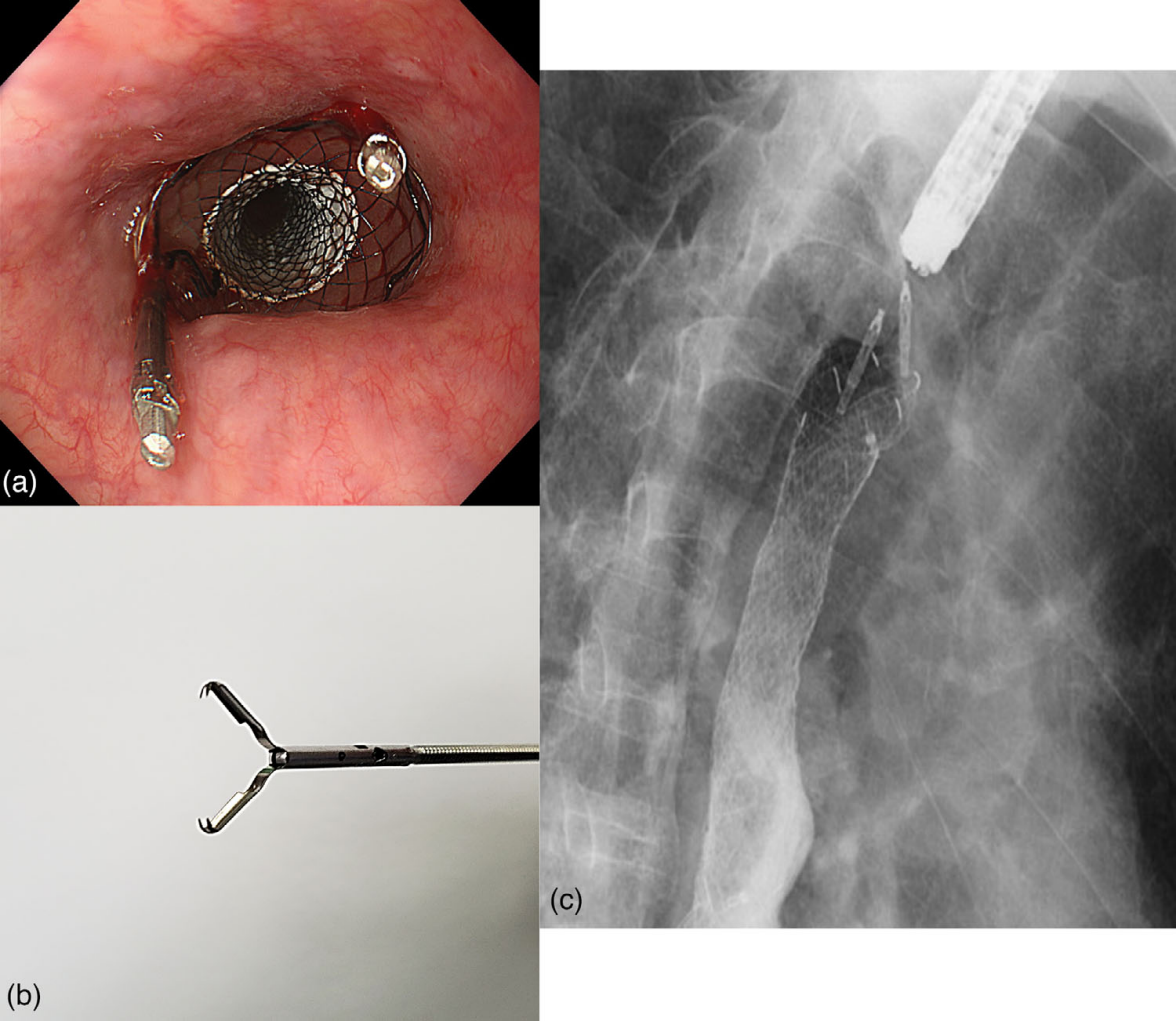
**Esophageal stent**. (a) The MANTIS Clip is used to grasp the mouth of the stent and the normal mucosa of the esophagus in concentric contralateral directions. (b) MANTIS Clip: a novel re‐openable clip with anchor prongs. (c) Gastrointestinal angiography is performed after esophageal stenting, with no contrast spillage into the bronchus.

**FIGURE 4:**
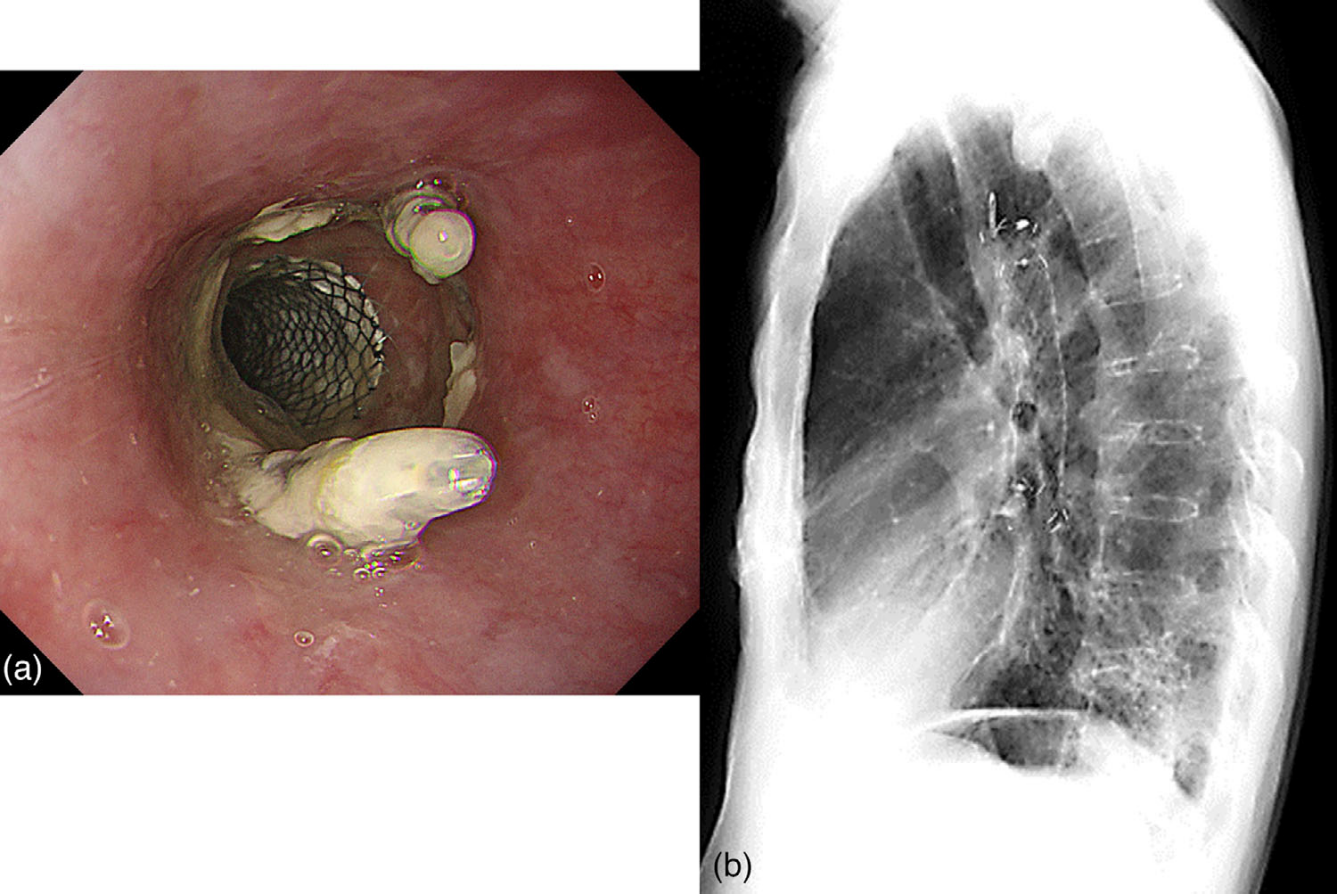
**Three weeks after esophageal stenting**. (a) Upper gastrointestinal endoscopy performed 3 weeks after esophageal stenting shows residual MANTIS Clip and no stent migration. (b) Chest X‐ray confirms no migration of the esophageal stent.

An 89‐year‐old man presented to a local physician with chest pain and dysphagia lasting 2 weeks. He had a history of hypertension, chronic obstructive pulmonary disease, and dementia. He had no history of alcohol consumption or smoking. Upper gastrointestinal endoscopy revealed a tumor in the thoracic esophagus, and the patient was suspected of having advanced esophageal cancer and was admitted to the Hiratsuka Kyosai Hospital. One week later, an upper gastrointestinal endoscopy performed at the same hospital revealed an elevated lesion with the upper wall of the mid‐thoracic esophagus as its main locus (Figure 1a). Scope passage was possible, and no fistulas were observed. Computed tomography showed that the tumor was in contact with the bronchi (Figure 1b). Histological examination revealed squamous cell carcinoma, leading to the diagnosis of esophageal squamous cell carcinoma (ESCC). Considering the patient's advanced age, we planned a treatment strategy involving radiotherapy. Radiotherapy consisted of 64.8 Gy in 27 fractions to treat the ESCC. Upper gastrointestinal endoscopy was performed 2 months after radiotherapy. Although the therapeutic effect on ESCC was evaluated as a complete response, a fistula was observed in the middle esophagus. Upper gastrointestinal endoscopy revealed that scope passage was possible without resistance (Figure 2a,b). Computed tomography revealed esophageal and bronchial traffic (Figure 2c), leading to the diagnosis of tracheoesophageal fistulas after radiotherapy for ESCC. Because the patient had difficulty with oral intake, we placed a partially covered self‐expandable esophageal metal stent (Niti‐S stent diameter, 18 mm; length, 120 mm) (Taewoong Medical). However, we considered the high risk of esophageal stent migration, as the esophageal stenosis was mild, and the scope was passable. Therefore, we used the MANTIS Clip to grasp the mouth of the esophageal stent and the normal mucosa of the esophagus and fixed the stent in two positions (Figure 3a,b). The esophageal stent was placed in 15 min and fixed in 6 min. Gastrointestinal angiography was performed after esophageal stenting, and no contrast spillage into the bronchus was observed (Figure 3c). The patient was capable of oral intake the day after the esophageal stent placement. Upper gastrointestinal endoscopy performed 3 weeks after esophageal stenting showed residual MANTIS Clip and no stent migration (Figure 4a). Chest radiography confirmed that the esophageal stent had not migrated (Figure 4b). The patient was subsequently transferred to a convalescent hospital with no complications associated with the esophageal stenting, and 55 days after transfer, he contracted sepsis and died.

## DISCUSSION

Patients with esophageal cancer with tracheoesophageal fistula may have esophageal stenting as a palliative treatment option, but complications include stent migration. Esophageal stenting is typically indicated for unresectable malignant esophageal stenosis, severe stenosis following radiotherapy, and tracheoesophageal fistulas.^1^, ^2^, ^6^, ^7^, ^8^ Although the incidence of stent migration in cases of severe stenosis is reported to be relatively rare, ranging from approximately 0% to 3.6%,^6^, ^7^, ^8^ tracheoesophageal fistulas are associated with a higher rate of stent migration than severe stenosis conditions.^2^ This makes the management of stent migration critical, as its occurrence can cause serious complications such as bleeding and perforation.^1^ In contrast, Singer et al. reported that fixation of esophageal stents was not associated with the prevention of migration with respect to migration of esophageal stents for complications after upper gastrointestinal surgery,^9^ and further research is warranted regarding methods of prevention of esophageal stent migration. This patient had a tracheoesophageal fistula following radiotherapy for ESCC. Since oral intake was difficult, esophageal stenting was considered a treatment option. However, because the esophageal stricture was mild, the risk of stent migration was very high, and prevention of stent migration was necessary.

Several esophageal stent fixation techniques have been reported to prevent esophageal stent migration.^3^, ^4^, ^5^ Singla et al. reported a combined endoloop and clip esophageal stent fixation technique,^4^ and Mudumbi et al. reported the efficacy and safety of the over‐the‐scope (OTSC) esophageal stent fixation for tracheoesophageal fistula and postoperative fistula and perforation.^5^ These reports represent a unique and reliable fixation technique. However, because of the complexity of the preparation and procedure, and the limited depths of penetration and closure strengths of the standard hemostatic clip, six hemostatic clips are used in endoloop esophageal stenting.^4^ Although the OTSC has strong grasping power and may be effective in preventing stent migration, even with fully covered esophageal stents,^5^ there are concerns regarding the inability to rechallenge once released, and that a safe method for retrieving the dislodged OTSC has not been established. Furthermore, the OTSC is very expensive and not available at all facilities.

The MANTIS clip is a new re‐openable clip with an anchor prong that provides strong tissue grasping force due to the presence of the anchor. Moreover, the MANTIS Clip is a retractable clip with quick rotation capability, allowing for simple manipulation and adjustment for precise tissue positioning. Recently, the MANTIS Clip has reportedly been used for suturing ulcers after endoscopic resection.^10^

To the best of our knowledge, this is the first report of a case of esophageal stent fixation using the MANTIS Clip. We performed several grasping and releasing procedures, changing the angle of the MANTIS Clip to determine the position where the stent could be firmly grasped to the normal mucosa of the esophagus. Finally, the MANTIS Clip was released on the contralateral side in a concentric circle to prevent migration. We were able to safely and quickly perform the procedure in a short time without complications. Thus, esophageal stent fixation using the MANTIS clip is relatively easy to perform; at the same time, it may improve the patient's quality of life by preventing esophageal stent migration and allowing oral intake. However, because the MANTIS clip is a closure device intended for the closure of perforations and mucosal defects, further case series are needed to investigate the safety and risks of esophageal stent migration. In this case, the partially covered esophageal metal stent was fixed using the MANTIS clip, which has an anchor protrusion but does not have as strong a grip as the OTSC, so the possibility of preventing migration of the fully covered esophageal stent should be investigated in the future.

In conclusion, esophageal stent fixation using the MANTIS Clip may be a potential treatment option for tracheoesophageal fistula.

## CONFLICT OF INTEREST STATEMENT

None.

## PATIENT CONSENT STATEMENT

Informed consent was obtained from the patient for the publication of this report.
